# Health Care Analytics With Time-Invariant and Time-Variant Feature Importance to Predict Hospital-Acquired Acute Kidney Injury: Observational Longitudinal Study

**DOI:** 10.2196/30805

**Published:** 2021-12-24

**Authors:** Horng-Ruey Chua, Kaiping Zheng, Anantharaman Vathsala, Kee-Yuan Ngiam, Hui-Kim Yap, Liangjian Lu, Ho-Yee Tiong, Amartya Mukhopadhyay, Graeme MacLaren, Shir-Lynn Lim, K Akalya, Beng-Chin Ooi

**Affiliations:** 1 Division of Nephrology Department of Medicine National University Hospital Singapore Singapore; 2 Department of Medicine Yong Loo Lin School of Medicine National University of Singapore Singapore Singapore; 3 Department of Computer Science School of Computing National University of Singapore Singapore Singapore; 4 Division of Endocrine Surgery Department of Surgery National University Hospital Singapore Singapore; 5 Department of Surgery Yong Loo Lin School of Medicine National University of Singapore Singapore Singapore; 6 Division of Paediatric Nephrology Department of Paediatrics National University Children’s Medical Institute Singapore Singapore; 7 Department of Paediatrics Yong Loo Lin School of Medicine National University of Singapore Singapore Singapore; 8 Department of Urology National University Hospital Singapore Singapore; 9 Division of Respiratory and Critical Care Medicine Department of Medicine National University Hospital Singapore Singapore; 10 Cardiothoracic Intensive Care Unit Department of Cardiac, Thoracic and Vascular Surgery National University Hospital Singapore Singapore; 11 Department of Cardiology National University Heart Centre Singapore Singapore

**Keywords:** acute kidney injury, artificial intelligence, biomarkers, clinical deterioration, electronic health records, hospital medicine, machine learning

## Abstract

**Background:**

Acute kidney injury (AKI) develops in 4% of hospitalized patients and is a marker of clinical deterioration and nephrotoxicity. AKI onset is highly variable in hospitals, which makes it difficult to time biomarker assessment in all patients for preemptive care.

**Objective:**

The study sought to apply machine learning techniques to electronic health records and predict hospital-acquired AKI by a 48-hour lead time, with the aim to create an AKI surveillance algorithm that is deployable in real time.

**Methods:**

The data were sourced from 20,732 case admissions in 16,288 patients over 1 year in our institution. We enhanced the bidirectional recurrent neural network model with a novel time-invariant and time-variant aggregated module to capture important clinical features temporal to AKI in every patient. Time-series features included laboratory parameters that preceded a 48-hour prediction window before AKI onset; the latter’s corresponding reference was the final in-hospital serum creatinine performed in case admissions without AKI episodes.

**Results:**

The cohort was of mean age 53 (SD 25) years, of whom 29%, 12%, 12%, and 53% had diabetes, ischemic heart disease, cancers, and baseline eGFR <90 mL/min/1.73 m^2^, respectively. There were 911 AKI episodes in 869 patients. We derived and validated an algorithm in the testing dataset with an AUROC of 0.81 (0.78-0.85) for predicting AKI. At a 15% prediction threshold, our model generated 699 AKI alerts with 2 false positives for every true AKI and predicted 26% of AKIs. A lowered 5% prediction threshold improved the recall to 60% but generated 3746 AKI alerts with 6 false positives for every true AKI. Representative interpretation results produced by our model alluded to the top-ranked features that predicted AKI that could be categorized in association with sepsis, acute coronary syndrome, nephrotoxicity, or multiorgan injury, specific to every case at risk.

**Conclusions:**

We generated an accurate algorithm from electronic health records through machine learning that predicted AKI by a lead time of at least 48 hours. The prediction threshold could be adjusted during deployment to optimize recall and minimize alert fatigue, while its precision could potentially be augmented by targeted AKI biomarker assessment in the high-risk cohort identified.

## Introduction

The clinical burden of acute kidney injury (AKI) worsens globally with the increasing complexity of cardiovascular diseases, anticancer therapy, and aging population [1-3]. AKI develops in 4% of patients admitted to our institution and involves more than 3000 patients annually [4]. A total of 39% of AKI cases develop during hospitalization following clinical deterioration and multiorgan dysfunction [4,5]. Additionally, 15% of patients who receive antimicrobials or chemotherapy of nephrotoxic potential develop drug-induced AKI [6,7]. Iodinated contrast administered for angiography contributes to AKI in 10% to 40% of patients with chronic kidney disease [8,9]. Once AKI develops in patients, however, the management remains supportive with control of its underlying triggers. AKI portends a poor patient prognosis with high mortality, prolonged hospitalization, and sustained deterioration of kidney function, with a significant risk of kidney failure in the long term [10,11].

Management strategies for high-risk patients may prevent AKI or reduce its downstream complications should AKI still develop. These measures must be implemented promptly, which requires the diagnosis of AKI in the subclinical phase, way before its onset. As the onset of AKI is highly variable during a patient’s stay, it is unclear how best to time biomarker surveillance for kidney injury concerning the patient’s clinical progress. The advent of electronic health records (EHRs) now provides us with real-time clinical data from routine patient care, built into millions of data points for analytics. These, along with AKI being defined by a numerical measure using serial serum creatinine, allow for an AKI prediction algorithm that is reproducible on a large scale. Machine learning with recurrent neural network–based techniques could improve the accuracy of analytics over traditional biostatistics [12]. These could be enhanced by capturing the relative feature importance temporal to AKI; that is, certain clinical covariates or trends (ie, features) would factor with increasing (or decreasing) importance in the time leading up to the onset of AKI. In this study, we would apply a novel machine learning technique that analyzes patient-related features in the form of routine hematology and biochemistry and their interaction with time to accurately predict AKI in hospitals by a lead time of 48 hours.

## Methods

### Dataset

The data source was our institution’s EHR in 2012, which recorded clinical and laboratory data from 68,832 case admissions in that year. Our institution is a 1200-bed academic hospital that provides complex tertiary care services including cardiothoracic surgery, transplantation, and cancer management. The Institutional Human Research Ethics Committee approved the study (NUHS-DSRB 2018/00169) and waived the need for informed consent given the use of deidentified data for analytics with secured institutional governance.

### Study Design and Participants

We performed an observational longitudinal study of the prospectively acquired EHR data from hospitalized patients in 2012. The exclusion criteria were (1) patients discharged within 48 hours of admission; (2) patients with community-acquired AKI, as inferred from onset of AKI within 48 hours of hospitalization [13]; (3) patients with stage 5 chronic kidney disease by Kidney Disease: Improving Global Outcomes (KDIGO) criteria, both dialysis, and nondialysis [14], inferred from diagnosis codes (Systematized Nomenclature of Medicine–Clinical Terms) for “end-stage kidney/renal disease,” an admission estimated glomerular filtration rate (eGFR) of less than 15 mL/min/1.73 m^2^ by Chronic Kidney Disease Epidemiology Collaboration equation [15], or procedural codes for peritoneal dialysis catheter insertion, arteriovenous access creation, or fistuloplasty; (4) patients with procedural codes for “**dialysis,” “**filtration,” or “**diafiltration” previously who failed to recover kidney function to a current admission eGFR of at least 30 mL/min/1.73 m^2^; or (5) patients with no available laboratory results for analytics.

### Definition of AKI

The binary event measure was AKI, as defined by the KDIGO 2012 criteria using serial serum creatinine levels during the index hospitalization [16]. These included the relative criterion of at least 1.5 times an increase in serum creatinine level within a 7-day window; the absolute criterion was an increase in serum creatinine of greater than 26.5 µmol/L (0.3 mg/dL) within 48 hours. The reference serum creatinine within the corresponding 7-day or 48-hour window for either criterion was taken as the baseline creatinine. The AKI-defining creatinine level and the extent of elevation over baseline were used to grade the initial KDIGO AKI staging severity. Creatinine was measured using the ADVIA 2400 (Siemens AG) enzymatic method traceable to isotope dilution mass spectrometry standard. We did not apply the oliguria criterion for AKI.

### Features Used for Analytics

**Figure 1:**
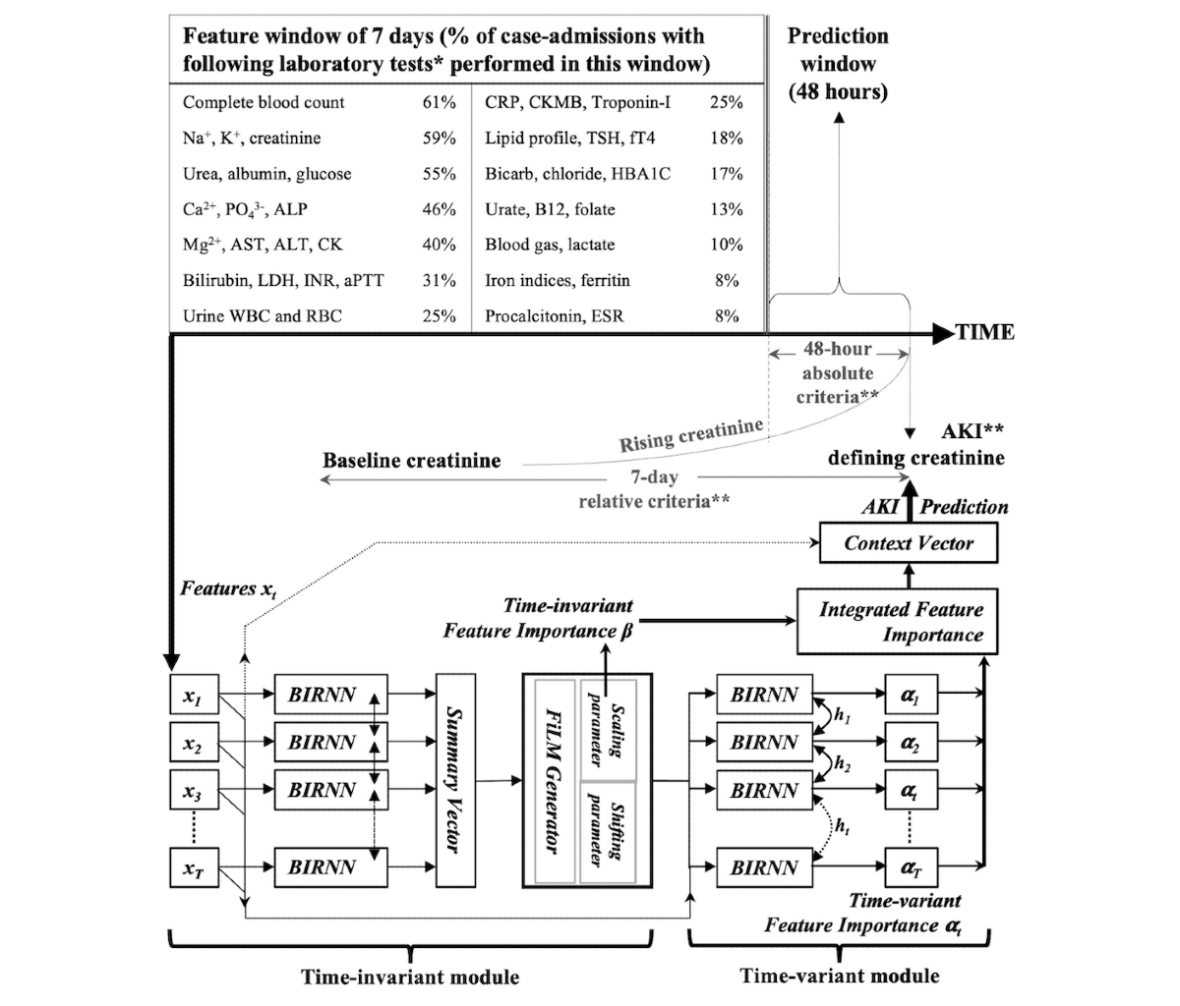
Prediction logic and features included in analytics. *: Serum biochemistry or hematology unless otherwise stated (eg, urine WBC and RBC); **: AKI defined by KDIGO criteria; x: features entered in model; t: time windows; β: time-invariant feature importance of which influence is shared across time windows; alpha-t: time-variant feature importance; ht: time-variant hidden representation; WBC: white blood cell; RBC: red blood cell; AKI: acute kidney injury; KDIGO: Kidney Disease: Improving Global Outcomes; BIRNN: bidirectional recurrent neural network; FiLM: feature-wise linear modulation.

Features (or covariates) were sourced from time-series laboratory results. The data source was our institution’s EHR, Computerized Patient Support System version 2 (Integrated Health Information System Pte Ltd). The results were integrated from comma-separated value files using common masked identifiers and ported onto our institution’s artificial intelligence discovery platform, an EHR analytic module. Data with the date and time stamps were selected as features to predict the event. These included all serial hematology, serum biochemistry, and urinary investigations (Figure 1). We did not include disease diagnosis codes or medication records.

### Analytics

Patient profile was compared between unique patients who developed AKI and those who did not. Parametric variables were reported as mean and standard deviation and compared using Student *t* tests; nonparametric variables were reported as median and IQR and compared using Wilcoxon rank-sum tests. Categorical variables were reported as frequency and percentage and compared using chi-square or Fisher exact tests where appropriate. A 2-tailed *P* value of <.05 was taken as the measure of statistical significance.

We sectioned the dataset by date and time for predictive analytics. Every case admission was taken as one sample. The first AKI episode that occurred in corresponding case admissions was analyzed. The AKI-defining creatinine served as the reference time point; the immediately preceding 48 hours was made the prediction window, and the feature window included the time up to 7 days before the prediction window (Figure 1). For case admissions with no AKI episodes, the corresponding reference time point would be the final serum creatinine level and likewise preceded by a 48-hour prediction window and a further 7-day feature window. Features performed within the feature window were used to predict AKI, a bivariate event, by a lead time of 48 hours. The feature window was further sectioned into daily serial time intervals for time-series modeling, temporal to the event. For each time interval, we averaged the values of the same feature, followed by normalization of the corresponding result *x* to generate a normalized *x*^1^ as the input for analytics, where *x*^1^ = [*x* – minimum(*x*)] / [maximum(*x*) – minimum(*x*)].

We proposed a novel time-invariant and time-variant (TITV) model to facilitate more accurate and interpretable analytics in AKI prediction based on the collaboration of 3 modules [17] (Figure 1). In the time-invariant module, an abstract representation was calculated with the data in the entire feature window, denoting each feature’s importance shared across time (ie, time-invariant feature importance). This time-invariant feature importance guided the modulation of input in the next module, the time-variant module. In this second module, we applied a bidirectional recurrent neural network to process sequential data and capture the dynamic behavior both forward and backward in time temporal to the event, as guided by the computed time-invariant feature importance from the time-invariant module. Additionally, we differentiated the influence of features across time windows leading to the event by applying the self-attention mechanism on top of the output of the bidirectional recurrent neural network; the output after the self-attention mechanism represents each feature’s importance in the corresponding time window (ie, time-variant feature importance in this time-variant module). Finally, in the prediction module, both the time-invariant and the time-variant feature importance were aggregated to calculate the final prediction (ie, risk of AKI). Meanwhile, the influence of each feature (in each time window) on the final prediction was also derived from the TITV model.

We performed a random shuffling of the entire cohort and arbitrarily partitioned the samples into 80% training, 10% validation, and 10% testing datasets. In the training process, we selected the hyperparameters that achieved the best performance in the validation dataset and applied them to the testing dataset for reporting of the experimental results [18-20]. We examined the reporting performance using the area under the receiver operating characteristic curve (AUC), as well as the respective sensitivity (recall) and positive predictive values (precision) that corresponded with the varying model prediction thresholds for AKI. Precision represents the proportion of predicted cases that truly had AKI; recall represents the proportion of actual AKI cases successfully identified by the prediction model. The AKI prediction threshold that provided the most optimal statistical balance between precision and recall was inferred by the highest computed F1 score. A high model recall gives rise, however, to more false positives (ie, poorer precision), and these permutations were further examined to demonstrate their clinical relevance to AKI diagnostics. These results were compared with the corresponding performance using traditional logistic regression and baseline recurrent neural network models. We applied zero imputation for missing data. Analysis was performed using Python (version 3.8.2, open source for Mac OSX).

## Results

### Patient Profile

**Figure 2:**
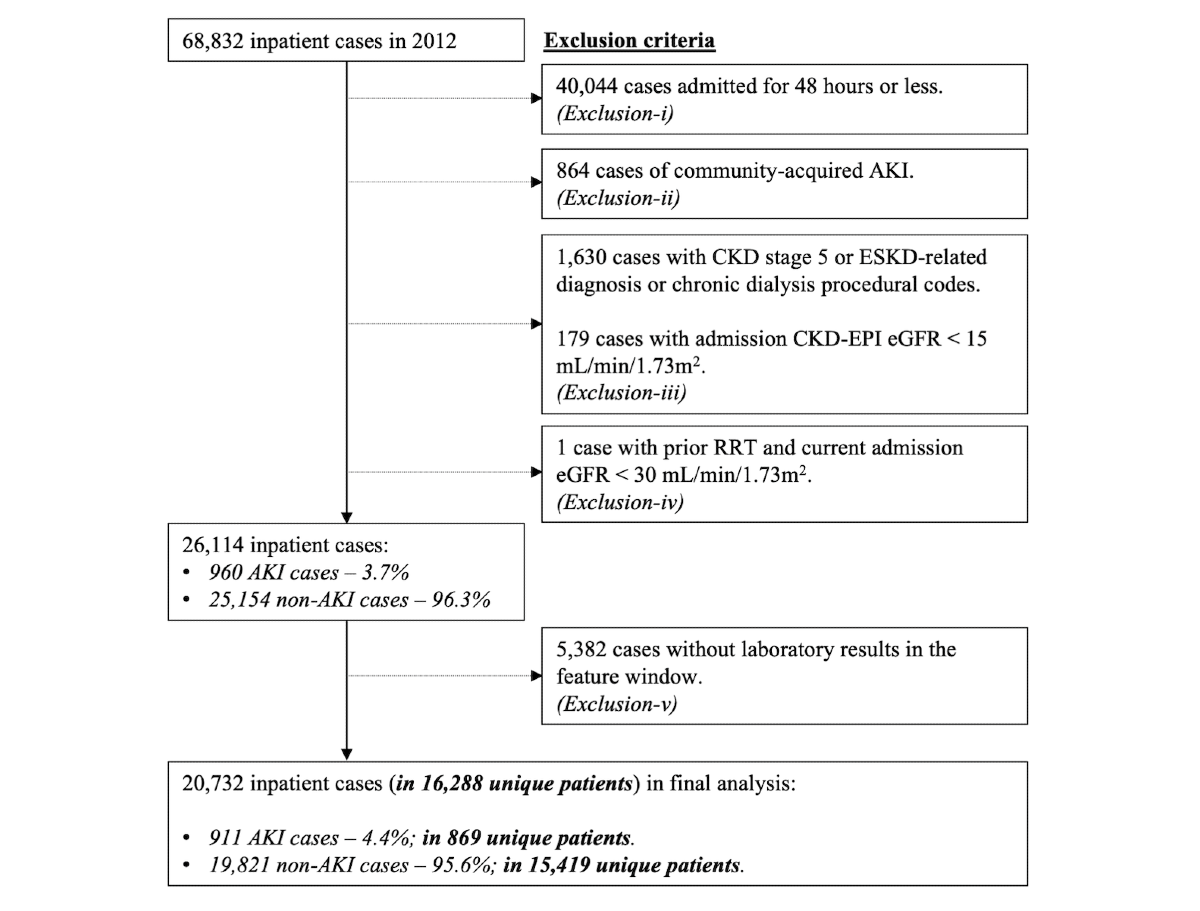
Study flow diagram. AKI: acute kidney injury; CKD: chronic kidney disease; CKD-EPI: Chronic Kidney Disease Epidemiology Collaboration equation; eGFR: estimated glomerular filtration rate; ESKD: end-stage kidney disease; RRT: renal replacement therapy.

We studied 20,732 case admissions in 16,288 unique patients, of which 1971 patients were younger than age 18 years (Figure 2). The mean age of the final cohort was 53 (SD 25) years, and 52.2 (8510/16,288) were males; 28.9% (4701/16,288) had diabetes, 35.0% (5699/16,288) had hypertension, 11.7% (1898/16,288) had ischemic heart disease, and 11.7% (1899/16,288) had either solid organ or hematological malignancy. Near half (7214/16,288, 44.3%) of patients had a baseline eGFR<90 mL/min/1.73 m^2^. More patients with AKI (258/869, 29.7%) had a baseline eGFR<60 mL/min/1.72 m^2^ compared to those without AKI (2738/15,419, 17.8%; *P*<.001; Table 1).

**Table 1:** Study profile and bivariate comparison between acute kidney injury and non–acute kidney injury patients.

Variables			Entire cohort (n=16,288)		AKI^a^ (n=869)		Non-AKI (n=15,419)		*P* value
Age, mean (SD), years			53 (25)		62 (22)		53 (26)		<.001
Male gender, n (%)			8510 (52.2)		480 (55.2)		8030 (52.1)		.08
**Comorbidities, n (%)**									
	Diabetes	4701 (28.9)		371 (42.7)		4330 (28.1)		<.001	
	Hypertension	5699 (35.0)		498 (57.3)		5201 (33.7)		<.001	
	Ischemic heart disease	1898 (11.7)		262 (30.1)		1636 (10.6)		<.001	
	Heart failure	1138 (7.0)		190 (21.9)		948 (6.1)		<.001	
	Cerebrovascular disease	757 (4.6)		73 (8.4)		684 (4.4)		<.001	
	Chronic liver disease	269 (1.7)		44 (5.1)		225 (1.5)		<.001	
	Solid organ malignancy	1556 (9.6)		140 (16.1)		1416 (9.2)		<.001	
	Hematological malignancy	343 (2.1)		52 (6.0)		291 (1.9)		<.001	
**Baseline kidney function**									
	Creatinine, µmol/L, median (IQR)	71 (54-92)		69 (46-108)		71 (55-91)		.05	
	eGFR^b^, mL/min/1.73 m^2^, median (IQR)	91 (67-109)		86 (55-110)		91 (68-109)		<.001	
	eGFR 90 or above mL/min/1.73 m^2^, n (%)	7628 (46.8)		400 (46.0)		7228 (46.9)		.65	
	eGFR 60 to <90 mL/min/1.73 m^2^, n (%)	4218 (25.9)		211 (24.3)		4007 (26.0)		.28	
	eGFR 45 to <60 mL/min/1.73 m^2^, n (%)	1328 (8.2)		101 (11.6)		1227 (8.0)		<.001	
	eGFR 30 to <45 mL/min/1.73 m^2^, n (%)	950 (5.8)		90 (10.4)		860 (5.6)		<.001	
	eGFR <30 mL/min/1.73 m^2^, n (%)	718 (4.4)		67 (7.7)		651^c^ (4.2)		<.001	
**AKI-related variables**									
	AKI-defining creatinine, µmol/L, median (IQR)	—^d^		122 (80-169)		—		—	
	Relative criterion (vs absolute), n (%)	—		651 (74.9)		—		—	
	AKI onset days from admission, median (IQR)	—		6 (3-10)		—		—	
**Serum biochemistry at AKI detection, median (IQR)**									
	Sodium, mmol/L	—		138 (135-142)		—		—	
	Potassium, mmol/L	—		4.1 (3.7-4.6)		—		—	
	Urea, mmol/L	—		11 (7-15)		—		—	
	Bicarbonate, mmol/L	—		24 (19-27)		—		—	
	Phosphate, mmol/L	—		1.23 (0.95-1.54)		—		—	
	Calcium, mmol/L	—		2.03 (1.89-2.17)		—		—	
	Chloride, mmol/L	—		105 (101-109)		—		—	
	Uric acid, µmol/L	—		384 (266-527)		—		—	
**Initial KDIGO^e^ AKI staging, n (%)**									
	Stage 1	—		701 (80.7)		—		—	
	Stage 2	—		125 (14.4)		—		—	
	Stage 3	—		43 (4.9)		—		—	
Total cumulative hospital days, median (IQR)			5 (3-10)		23 (13-44)		5 (3-9)		<.001
Hospital days per admission, median (IQR)			5 (3-8)		14 (8-26)		5 (3-7)		<.001

^a^AKI: acute kidney injury.

^b^eGFR: estimated glomerular filtration rate by Chronic Kidney Disease Epidemiology Collaboration equation.

^c^A total of 1446 non-AKI patients had missing baseline eGFR.

^d^Not applicable.

^e^KDIGO: Kidney Disease: Improving Global Outcomes.

### Evaluation Outcomes

AKI developed during 4.4% (911/20,732) of case admissions in 869 unique patients at a median of 6 (IQR 3-10) days from admission; 74.9% (651/869) of AKI patients were diagnosed based on KDIGO relative criterion, and 80.7% (701/869) were of initial KDIGO stage 1 in severity. Patients who developed AKI were older with more comorbidities including diabetes, hypertension, cardiovascular diseases, chronic kidney disease, chronic liver disease, and cancers compared with non-AKI patients (all *P*<.001). The median hospital days per admission and cumulatively in 2012 in AKI patients versus those without were 14 (IQR 8-26) days versus 5 (IQR 3-7) days, and 23 (IQR 13-44) days versus 5 (IQR 3-9) days, respectively (all *P*<.001; Table 1).

### Analytics for AKI Prediction in the Hospital

The 7-day feature window was divided into daily time windows, giving a total of 7 time windows and 709 features in the analysis. Figure 1 shows the laboratory variables included in the feature window in order of their corresponding test prevalence by categories. Complete blood count was the most common investigation, performed in 61.3% (12,709/20,732) of all case admissions in the analysis; this was followed by serum electrolytes, urea, and creatinine at 46% to 59%, and liver function markers at 30% to 41%. In comparison, acid-base parameters and serum lactate contributed less (2146/20,732, 10.4%) to the analysis.

**Figure 3:**
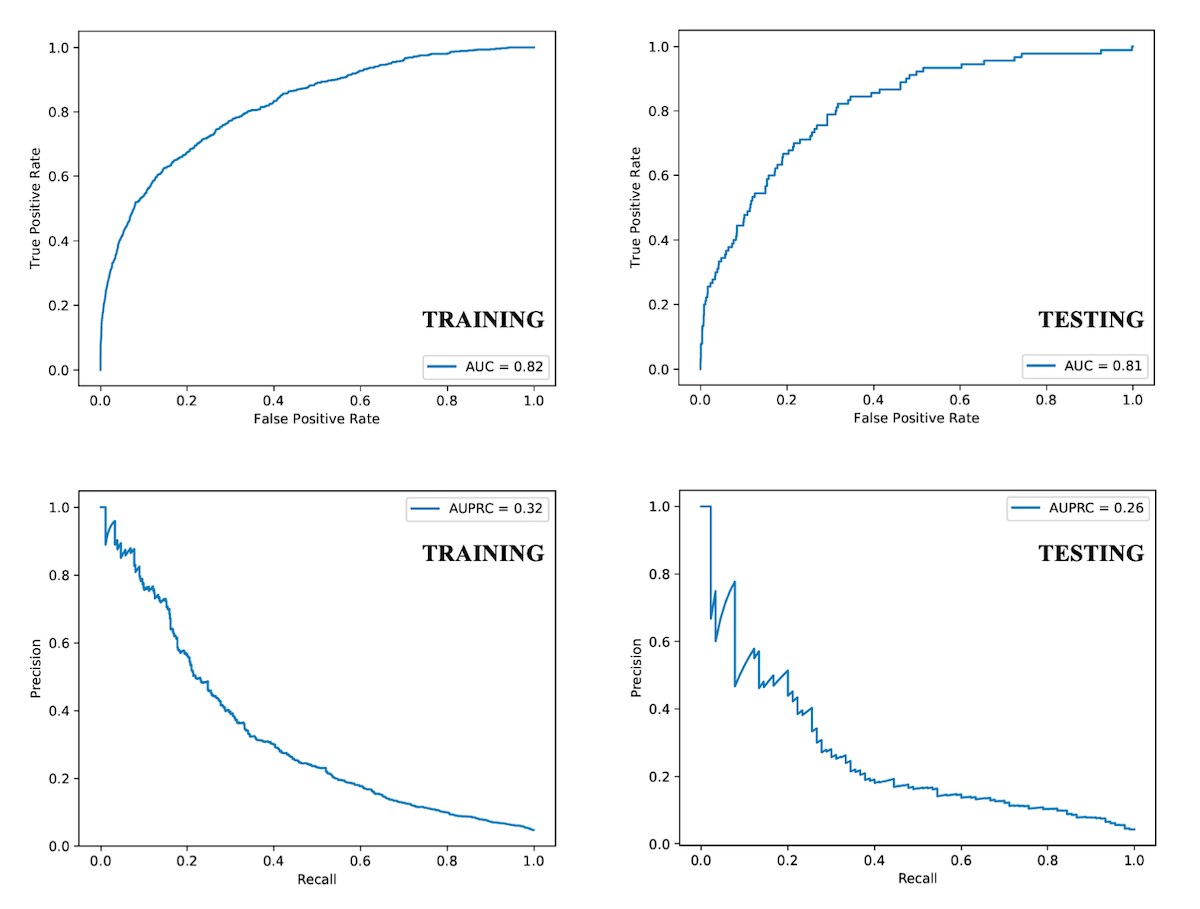
Area under receiver operating characteristic and area under precision-recall curves of training and testing datasets. AUC: area under receiver operating characteristic curve; AUPRC: area under precision-recall curve.

The cohort was partitioned into the training (16,585 cases), validation (2073 cases), and testing (2074 cases) datasets; AKI rates in the 3 datasets were 4.5%, 3.9%, and 4.3%, respectively. Table 2 summarizes the AUC of respective analytic modules in the final testing dataset as well as the precision and recall corresponding with the AKI prediction threshold with the highest F1 score. The AUC for AKI prediction by the multivariate logistic regression and recurrent neural network/time-series models were 79% and 80%, respectively. The AUC was 81% after we applied the TITV module with comparable precision and recall compared with the former models; these and the highest F1 score were achieved at an AKI prediction threshold between 15% and 20%. The respective AUCs and corresponding area under precision-recall curves for the training and testing datasets are illustrated in Figure 3.

Table 3 shows the breakdown in our TITV module precision and recall according to the varying probability thresholds for AKI prediction.

**Figure 4:**
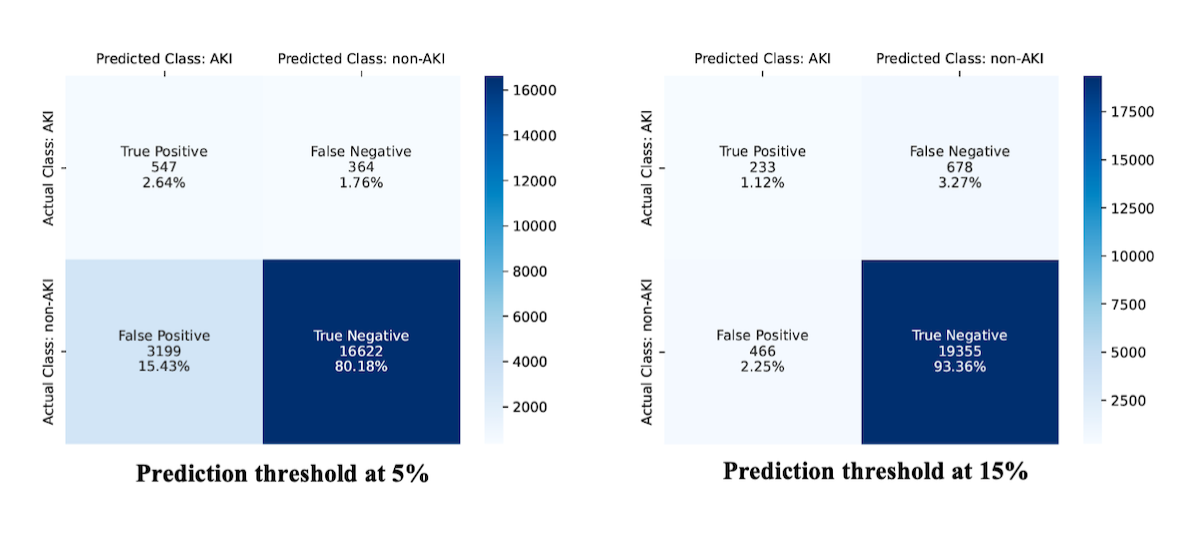
Confusion matrix plots with acute kidney injury prediction thresholds at 5% and 15%.

A low prediction threshold detected a very high number of predicted AKI cases that scored high in recall but poor in discrimination between true and false positives. Conversely, a high prediction threshold detected a low number of predicted AKI cases but with high precision. A 15% AKI probability threshold implied that 699 cases were predicted to be diagnosed with AKI; 33.3% (233/699) of predicted cases did subsequently develop AKI, while 25.6% (233/911) of eventual AKI cases were successfully predicted. Reducing the probability threshold to 5% led to 3746 predicted AKI cases with much higher false positives but with successful prediction of 60.0% (547/911) of eventual AKI cases. Figure 4 illustrates the confusion matrix plots at AKI prediction thresholds of 5% and 15%. Further details on TITV performance metrics are provided in Table 4.

**Figure 5:**
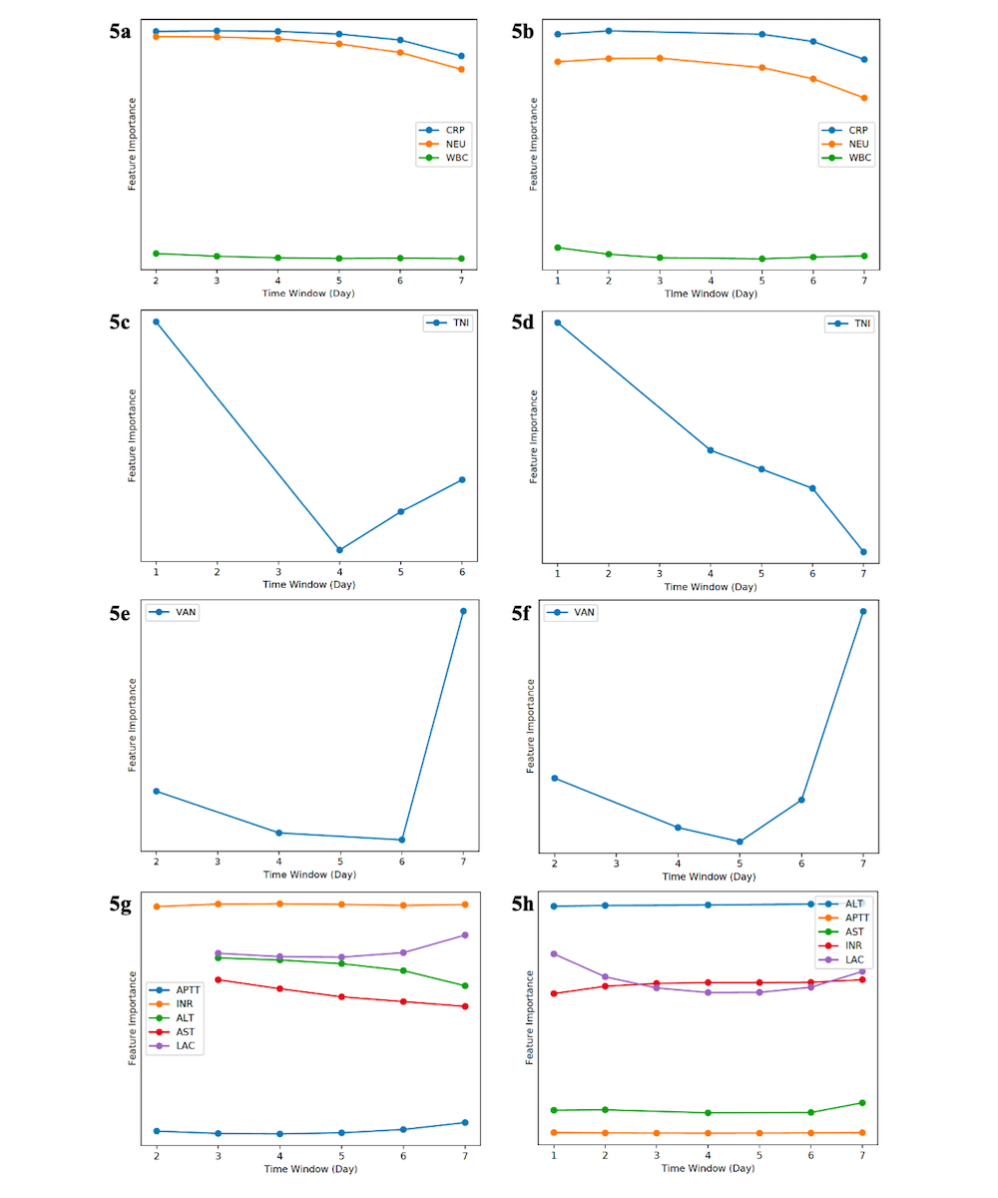
Case examples of relative feature importance in acute kidney injury (AKI) prediction. Time-window: refers to feature window of 7 days in AKI prediction; Y-axis: features highly associated with AKI would rank high in relative feature importance; a-b: C-reactive protein, neutrophils featured prominently over days, which suggested infection and inflammation were associated with subsequent AKI; c-d: troponin-I featured prominently initially, which suggested cardiac disease in association with AKI, although its relative importance waned in subsequent days; e-f: vancomycin levels rose in feature importance proximate to AKI, which strongly suggested vancomycin nephrotoxicity; g-h: lactate, liver enzymes, international normalized ratio, and activated partial thromboplastin time featured strongly, which suggested hepatic or multiorgan dysfunction in association with evolving AKI.

In addition, our TITV model generated representative interpretation results specific to each AKI case. Figure 5 illustrates the relative feature importance to AKI in 8 case examples, which demonstrated the range of inflammatory, cardiac, drug-specific, or hepatic functional markers in association with AKI, specific to each case. The source codes for our predictive algorithm are available online [21].

**Table 2:** Acute kidney injury predictive performance in the testing dataset with optimized F1.

Model	Precision^a^	Recall^b^	F1^c^	AUC^d^ (95% CI)
Logistic regression	0.274	0.189	0.224	0.789 (0.752-0.827)
RNN^e^ (GRU^f^)	0.286	0.222	0.250	0.800 (0.764-0.836)
BRNN^g^ (BGRU^h^)	0.309	0.233	0.266	0.797 (0.761-0.833)
Proposed TITV^i^ model	0.397	0.256	0.311	0.814 (0.780-0.848)

^a^Precision: true positive / (all cases predicted at risk of acute kidney injury).

^b^Recall: true positive / (all cases that eventually developed acute kidney injury).

^c^F1 score: 2 × [(recall × precision) / (recall + precision)].

^d^AUC: area under receiver operating characteristic curve.

^e^RNN: recurrent neural network.

^f^GRU: gated recurrent unit.

^g^BRNN: bidirectional recurrent neural network.

^h^BGRU: bidirectional gated recurrent unit.

^i^TITV: time-invariant and time-variant feature importance.

**Table 3:** Varied acute kidney injury prediction thresholds on time-invariant and time-variant model performance metrics.

Threshold^a^ to predict AKI^b^ (%)	Precision^c^	Recall^d^	F1^e^	Predicted AKI cases by model, n	True positive AKI cases, n
5	0.146	0.600	0.235	3746	547
10	0.252	0.333	0.287	1204	304
15	0.333	0.256	0.289	699	233
20	0.500	0.200	0.286	364	182
25	0.480	0.133	0.209	253	121
30	0.556	0.111	0.185	182	101

^a^Probability threshold to define predicted AKI versus no risk of AKI (ie, positive/negative class prediction). A low threshold risks over-detection and alert fatigue, which corresponds to poor precision. A high threshold risks missing true AKI cases, which corresponds to poor recall.

^b^AKI: acute kidney injury.

^c^Precision: true positive / (all cases predicted at risk of AKI).

^d^Recall: true positive / (all cases who eventually developed AKI).

^e^F1 score: 2 × [(recall × precision) / (recall + precision)].

**Table 4:** Model performance metric with time-invariant and time-variant prediction thresholds at 5% and 15%.

		True AKI^a^ cases	No AKI	Subtotal
**5% prediction threshold**				
	TITV^b^ predicted (positive)	547	3199	3746
	TITV predicted (negative)	364	16,622	16,986
	Subtotal	911	19,821	20,732
**15% prediction threshold**				
	TITV predicted (positive)	233	466	699
	TITV predicted (negative)	678	19,355	20,033
	Subtotal	911	19,821	20,732

^a^AKI: acute kidney injury.

^b^TITV: time-invariant and time-variant module.

## Discussion

### Principal Findings

We have used structured but heterogeneous biochemical data from 20,732 case admissions in the prediction of hospital-acquired AKI by a 48-hour lead time. We enhanced the recurrent neural network model with a novel analytic module that took into account the temporal interactions in serial laboratory parameters that inferred disease trajectory leading up to AKI [17]. At the optimal statistical operation point as indicated by the highest F1 score (Table 2), our module generated 3 false positives for every 2 true AKI cases, and clinicians would need to act on just 600 predicted AKI alerts of 20,732 case admissions yearly; however, 3 of 4 true AKI cases would be missed. It may be more desirable for our module to successfully predict at least 3 of 5 true AKI cases, but this is counterbalanced by 6 false positives for every 1 true AKI case, and more than 3000 predicted AKI alerts yearly (Table 3). We suggest that our AKI prediction threshold should be low to identify more patients at risk of AKI daily. This narrows the entire hospital cohort to a more manageable patient number for closer monitoring, in whom further assessment could be augmented by AKI biomarkers to reduce false positives [22]. These include urinary clusterin, kidney injury molecule–1, tissue inhibitor of metalloproteinase–2, and insulin-like growth factor binding protein–7, for which levels rise in 12 to 48 hours before a significant rise in serum creatinine [7,23].

### Comparison With Prior Work

Our methodology differs from machine learning techniques that used a quasi-random selection of variable prediction points relative to AKI [24]. It resembles models that adopted structured feature and prediction windows relative to AKI that facilitate the deployment of our prediction algorithm in real time [25]. Importantly, we expanded the prediction window to a minimum of 48 hours. Such improved lead time may be necessary for any AKI preventive strategies to make a meaningful change in clinical outcomes. Preemptive interventions may include more detailed patient reviews, timely treatment of infections, precise volume management [26], preferred use of balanced electrolyte over chloride-rich solutions [27], admission to high-dependency or intensive care unit for detailed monitoring, and reduction in or cessation of nephrotoxic medications [28]. These measures, when implemented in a timely fashion and supported by a responsive EHR platform for AKI alerts, may reduce the hospital days and AKI duration in affected patients [29,30].

The performance of any analytic module depends strongly on the appropriate feature selection. Our model was built from objective laboratory test results that would be similar in data structure across institutions [31]. Our algorithm used routinely performed hematology and biochemistry without disease diagnosis codes; these included complete blood count, common electrolytes, acid-base parameters, and liver and cardiac enzymes, and these remain relevant for current AKI prediction even with the changing health care landscape. As our analysis was limited to available investigations performed before a mandatory 48-hour prediction window, the laboratory indices analyzed in the feature window might not be comprehensive. This could compromise the model performance, and the prediction should otherwise improve with features performed at higher frequency and more proximate to AKI [25,32]. Despite this, we demonstrated an AUC that exceeded 80% for AKI prediction in our testing dataset. Certain indices like blood gas, serum lactate, cardiac enzymes, and drug levels should increase in frequency and importance toward the onset of AKI, since AKI serves as a marker of clinical deterioration from nosocomial infections, decompensated cardiovascular diseases, major surgery, or nephrotoxicity [33,34]. Varying significance of these time-sensitive features in association with evolving AKI may be seen among subsets of patients with sepsis, cardiac failure, or cardiac surgery [35-37]. Our TITV module can provide patient-level interpretation of the feature importance, as suggested by our representative interpretation results in unique AKI case examples (Figure 5). These could provide insightful patient-specific trends to aid the evaluation of AKI etiology [17].

### Strengths and Limitations

Our study has several strengths but is not without limitations. We have studied a large and diverse population with a comprehensive range of medical and surgical conditions not confined to critical care, which improves the generalizability of our analytic module to hospital practice. We excluded patients with more advanced chronic kidney disease, and our 4% incidence of AKI in the hospital was lower than the 8% reported in prior studies that used similar EHR methods [33,38]. The lack of precise urine output in ward patients could reduce the model accuracy, but oliguria often develops in 24 hours proximate to AKI and may not fulfill our requirement for a 48-hour prediction window. We have normalized the variables for standardized comparison across different tests. Our novel TITV module provided fine-grained interpretability of the prediction results and achieved accurate prediction simultaneously; this facilitates high-quality health care analytics. Being single center in nature, our AKI prediction module needs to be applied and validated in external health care systems to demonstrate reproducibility. The prediction algorithm could be ported to run on platforms that use similar EHR data architecture, but this naturally limits its deployment to institutions with available technology. Nevertheless, our model could be applied for rolling AKI predictions daily if coupled with a real-time feed of laboratory data. While forward application of the algorithm would naturally encounter model degradation due to concept drift, novel techniques could achieve concept drift detection, understanding, and further adaption from contemporaneous data [39,40]. Furthermore, our algorithm was based on laboratory test results less subjected to case-mix shift over time as compared with disease diagnoses or medication records [41]. We had used zero imputation for missing data, unlike the previously described method of imputing preexisting values in time or median value [38]; zero imputation has been widely adopted in machine learning techniques and has achieved state-of-the-art performance in analytics [42,43]. Finally, the subcohort with “false-positive AKI” might be analogous to that of patients with subclinical AKI that may also be associated with adverse long-term outcomes; these were not explored in our study.

### Conclusions

We have presented a feasible and enhanced EHR analytic module that captures time-sensitive interactions in laboratory investigations and predicts hospital-acquired AKI by a 48-hour lead time. The AKI prediction threshold could be varied to allow the clinically relevant balance in model precision, recall, and predicted AKI numbers that are compatible with patient service load in health care institutions. With a compromised precision in favor of the better recall, our model serves to risk stratify ward patients for detailed clinical or biomarker assessment for true AKI risk. Its real-time deployment is expected to greatly facilitate our upstream efforts to prevent AKI or its complications in hospitalized patients.

## Abbreviations

AKI: acute kidney injury
AUC: area under the receiver operating characteristic curve
eGFR: estimated glomerular filtration rate
EHR: electronic health record
KDIGO: Kidney Disease: Improving Global Outcomes
TITV: time-invariant and time-variant
